# Prioritizing health care workers and first responders for access to the COVID19 vaccine is not unethical, but both fair and effective – an ethical analysis

**DOI:** 10.1186/s13049-021-00886-2

**Published:** 2021-06-04

**Authors:** Bjorg Thorsteinsdottir, Bo Enemark Madsen

**Affiliations:** 1 Department of Medicine, Division of Community Internal Medicine, Program in Bioethics, Kern Center for the Science of Health Care Delivery, Mayo Clinic, 200 First Street SW, Rochester, MN 55905 USA; 2 Department of Emergency Medicine, Mayo Clinic, Rochester, MN 55905 USA

**Keywords:** Health care worker, Resource allocation, Vaccine, COVID19, Priority

## Abstract

The Nordic countries have differed in their approach as to how much priority for COVID19 vaccine access should be given to health care workers. Two countries decided not to give health care workers highest priority, raising some controversy. The rationale was that those at highest risk of dying needed to come first. However, when it comes to protecting those at the highest risk of dying from COVID19, their needs and vulnerabilities need to be considered more broadly than just in terms of the individual protection that vaccination will afford them. Likewise, when considering whether to prioritize health care workers for the vaccine, their crucial role in keeping the health care system operational, and right to a safe work environment need to be factored in. Below we review several ethical arguments for why frontline health care workers and first responders should receive priority access to the COVID19 vaccine.

## Background

As nations work to secure enough COVID19 vaccine supplies for their populations they also weigh how to distribute the vaccine fairly and effectively. Several primary guideposts govern allocation decisions in most countries. First the imperative to protect those at highest risk of dying, second the need to cover those at highest risk of exposure and complications, and finally the imperative to secure essential services and enable society to return to normal functioning. Many countries have placed their health care workers (HCW) high on the priority list for vaccine access. Two Nordic countries, stood out in their initial declarations of an intent not to prioritize HCW, with government officials going as so far as declaring it unethical to prioritize health care workers for vaccine access [1]. Although the approach has since been revised [2, 3], these statements still deserve scrutiny. This article provides an opposing ethical rationale.

People can approach justice from many different angles. This article focuses on the ethos largely governing the Nordic health care systems, namely solidarity, meeting the needs of all individuals irrespective of social status, social worth or ability to pay while keeping in mind the greater societal good [4]. Indeed these values are espoused in the Danish ethical counsel’s recommendations for vaccine prioritization [5]. This article is moreover written assuming the realities of the Nordic socialized health care systems and thus some arguments may not apply to other settings. Below we review several ethical arguments for why frontline health care workers and first responders should be prioritized for the vaccine.

## Main text

### Vaccinating HCW will protect those at highest risk from getting infected

During the lockdown of long-term care facilities, HCW have become the primary contact of residents with the outside world and thus the most likely to bring COVID19 to the facilities. This is particularly concerning given the relatively high risk of asymptomatic spread. Influenza vaccinations of health care workers are recommended by the WHO to protect patients [6]. With COVID19 five times more lethal than the flu, the imperative to vaccinate HCW to decrease exposure risk is all the more compelling [7]. Some have argued that vaccinating the young to protect the old may be more effective than vaccinating the institutionalized elderly, given waning immune response at advanced age [8].

### The institutionalized old and disabled patients rely on HCW for their care

High infectious rates at nursing homes that have been hard hit by COVID19 outbreaks have led to breakdown in care, leading to neglect and suffering and putting lives at risk [9]. Thus prioritizing HCW in long term care settings would help ensure adequate staffing and patient safety.

### HCW and first responders are essential in keeping the health care system running

High risk patients who get ill from COVID19 and require emergency and hospital care rely on highly trained and skilled HCW to deliver life-saving treatments. With many hospitals operating at or over capacity during the pandemic they can ill afford losing staff to COVID19 illness, at times with additional protective quarantine for co-workers. Therefore, vaccinating those HCW who take care of COVID patients, protects those who fall ill during the pandemic from lack of access to hospitals and life-saving treatments.

### HCW are at an increased risk for infection despite personal protective equipment (PPE)

HCW the world over have faced shortages of adequate PPE [10]. Despite the widespread use of PPE and pre-visit symptom screening, HCW are almost twice as likely to contract COVID19 than the general population and they are seven times more likely to suffer severe COVID19 disease [11, 12]. While PPE has proven quite effective in decreasing disease transmission, the disease can be airborne, potentially leading to infections despite PPE. HCW have a right to a safe work environment [13]. Therefore, when better protection through a vaccine becomes available, they should get priority access to it.

### Not all HCW are young and healthy

HCW and their family members like others can have conditions that put them at higher risk. Health care systems have not always been able to accommodate high risk workers with lower risk job assignments. Additionally, there are many examples of retirees returning to the work force to help in the current crisis. The primary ethical principle to do no harm, renders it unethical to continue to put these vulnerable HCW at risk for infection when a vaccine is available.

### Health care workers deserve reciprocity for putting their lives on the line for the lives of others

Since the beginning of the pandemic, while stories of a deadly plague circulated, PPE scarcity loomed, and deaths of HCW caught international attention, physicians, nurses and others have continued to show up to take care of COVID19 infected patients. The stress of taking care of a teammate is well recognized. In military and natural disaster triage, this translates into an absolute priority for taking care of a teammate who is down [14]. The social contract needs to include reciprocity so that HCW trust that they will be taken care of as they risk their lives taking care of others.

### Health care workers are essential in ensuring the success of the vaccination scheme

Currently available vaccines require expert handling and pre-vaccination health screening. Staffing can be a rate limiting factor for vaccine distribution success. Close encounter with hundreds of people in a short time frame also exponentially increases exposure risk for vaccination staff. Therefore failing to adequately provide staff with protection through a highly effective vaccine is ethically questionable. There is also risk of vaccine diversion if HCW do not feel that their safety is given adequate priority [15].

## Conclusion

There are strong ethical justifications for prioritizing health care workers for the COVID19 vaccine. In addition to honoring their right to a safe work environment, their vaccination also protects their patients and helps keep the health care system operating during a time of dire need. Health care workers, who despite a risk to their lives, continue to show up and serve their fellow citizens deserve to be kept safe in this and future pandemics.

## Data Availability

N/A no data to make available beyond the reference list.
